# Osteoblastoma in the parietal bone in elderly: rare tumor in the calvarium

**DOI:** 10.11604/pamj.2021.39.158.29766

**Published:** 2021-06-29

**Authors:** Inas El Kacemi, Miloudi Gazzaz

**Affiliations:** 1 Service de Neurochirurgie de l´Hôpital Militaire d´Instruction Mohammed V-Rabat, Faculté de Médecine et de Pharmacie, Université Mohammed V de Rabat, Rabat, Maroc

**Keywords:** Elderly, osteoblastoma, osteoma osteoid, parietal bone

## Image in medicine

Osteoblastomas are rare bone-forming tumors that may be locally aggressive, they account for 1-3% of all primary bone tumors with the majority presenting in young adults between their second and third decades of life. This tumor predominantly affects the vertebral column and long tubular bones. Skull involvement is markedly less frequent, and particularly parietal bone involvement is extremely rare, osteoblastoma is a rare tumor older than 50 years of age. Here, we describe a case of a 70-year-old woman who presents with headache and swelling of the vault of the skull, computed tomography (CT) of the head, which demonstrated an expansible, mixed lucent and sclerotic mass in the parietal bone with a suggestion of osteoid matrix calcifications. In its largest extent, the mass measured up to 5.7 cm, and magnetic resonance imaging (MRI) revealed a mass lesion in parietal bone with low to intermediate T1 signal intensity and intermediate to high T2 signal intensity. The patient underwent surgical resection with complete removal of the parietal mass. Histopathological examination revealed an osteoblastoma. Osteoblastoma is a rare tumor older than 50 years of age, but it should be considered in the differential diagnosis of bone lesions of the calvarium in adulthood and in the elderly, to avoid a delay in the treatment.

**Figure 1:**
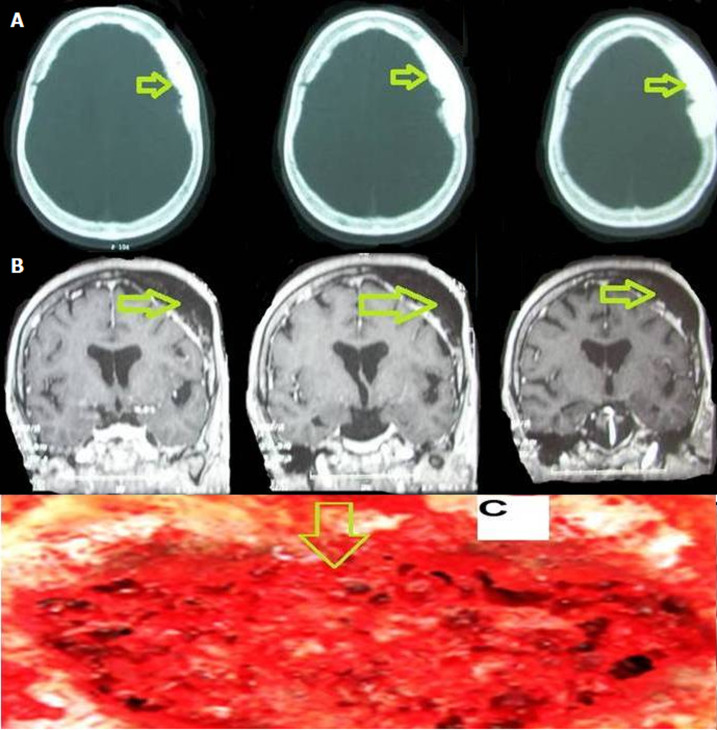
A) computed tomography (CT) of the head which demonstrated a mass with a bone density, which infiltrates the parietal bone; B) cerebral MRI in frontal section sequence T1 which demonstrated a mass lesion in parietal bone with low to intermediate T1 signal intensity; C) intraoperative image showing the lesion

